# Gut-brain axis: a revolutionary paradigm in the pathogenesis and treatment of ischemic stroke

**DOI:** 10.3389/fneur.2026.1793860

**Published:** 2026-08-03

**Authors:** Yu Gou, Hong Bai, Xingshun Zhou, Cong Huang

**Affiliations:** No. 926 Hospital, Joint Logistics Support Force of PLA, Kaiyuan, China

**Keywords:** gut microbiota, gut-brain axis, ischemic stroke, microbial metabolites, neuroinflammation, stroke intervention

## Abstract

Ischemic stroke is a devastating neurological disorder. Despite advances in acute reperfusion therapy, patients still face high risks of poor functional recovery and stroke recurrence, highlighting the urgent need to explore new pathological mechanisms and therapeutic targets. In recent years, the Gut-Brain Axis—a bidirectional communication network connecting the brain, gut, and gut microbiota—has provided a novel framework for understanding stroke pathophysiology. This review systematically summarizes how gut microbiota correlates with the progression of major stroke risk factors including hypertension and atherosclerosis, and explicitly distinguishes that most human observational data only support correlative associations, while causal evidence is largely limited to preclinical animal models. We further clarify the two-way communication pathway after stroke: cerebral ischemia impairs intestinal barrier function and causes microbial imbalance through autonomic and inflammatory responses; in turn, disturbed gut microbiota and metabolites correlate with aggravated brain damage and hindered repair via immune, metabolic, and neural signaling, mainly by regulating neuroinflammation and controlling key metabolites such as short-chain fatty acids and trimethylamine N-oxide. Based on these mechanisms, we comprehensively assess preclinical and clinical evidence of microbiota-targeted interventions, including dietary modification, probiotics, and fecal microbiota transplantation. We emphasize that existing clinical evidence is mostly derived from small-sample, single-center trials with inconsistent outcomes, and large-scale multicenter sham-controlled randomized controlled trials are mandatory before any routine clinical application can be recommended. We also discuss key challenges such as distinguishing causal relationships from correlative observations, bridging preclinical translational gaps, and addressing inter-individual heterogeneity across patient populations, and propose future translational research directions. Targeting the Gut-Brain Axis represents a promising preclinical systemic strategy for stroke prevention, acute care, and long-term rehabilitation, yet robust human validation is still lacking. This approach marks a fundamental paradigm shift in the field of stroke research, though overoptimistic clinical translation expectations should be avoided given the substantial unresolved translational limitations.

## 1 Introduction

Ischemic stroke ranks among the leading neurological causes of mortality and long-term disability worldwide, imposing a substantial health and economic burden on society (1). Although milestone advances have been made in acute reperfusion therapy—including intravenous thrombolysis and mechanical thrombectomy—these interventions are constrained by narrow therapeutic time windows and are applicable to only a subset of patients (2, 3). Moreover, even when successful recanalization is achieved, a significant number of patients still experience poor outcomes due to reperfusion injury, secondary neuroinflammation, and long-term complications (4). In the realm of secondary prevention, antiplatelet agents, antihypertensives, and statins form the cornerstone of treatment; however, the risk of stroke recurrence remains considerable (5). These unmet clinical needs highlight the urgency of exploring novel pathophysiological mechanisms underlying ischemic stroke and developing innovative treatment strategies accordingly.

Traditionally, ischemic stroke was viewed as a focal cerebrovascular event dominated by ischemia–hypoxia and subsequent cell death. Emerging evidence, however, reveals that cerebral ischemia instantaneously ignites a precisely choreographed, systemic immune–inflammatory cascade that reaches far beyond the brain to perturb multiple distant organs, notably the gut (6, 7). This conceptual reframing of stroke as a systemic disorder has re-oriented research toward previously unexplored pathophysiological dimensions and inter-individual heterogeneity in recovery.

Central to this shift is the gut–brain axis, an integrated, bidirectional communication network linking the central, enteric and neuroendocrine systems with the gut microbiota and their bioactive metabolites (8, 9). The microbiota, the largest symbiotic ecosystem in humans, continuously exchanges immunological, neural, endocrine and metabolic signals with the brain to preserve homeostasis (10). After stroke, this equilibrium collapses. On the one hand, ischemic insult activates the autonomic nervous system and the hypothalamic–pituitary–adrenal axis, compromising intestinal barrier integrity (“leaky gut”), provoking dysbiosis and fostering bacterial translocation that amplifies systemic inflammation (11, 12). On the other hand, pre-existing dysbiosis and specific microbial metabolites are associated with stroke risk, infarct severity and long-term functional recovery. Beneficial metabolites include short-chain fatty acids (SCFAs), while harmful metabolites include trimethylamine N-oxide (TMAO) (13, 14).

This narrative review was constructed via a structured comprehensive literature search without formal PRISMA systematic review methodology. We systematically searched PubMed, Embase, and Web of Science from inception to December 2025 using keywords: “gut-brain axis,” “ischemic stroke,” “gut microbiota,” “microbial metabolites,” “stroke intervention,” “neuroinflammation.” Included studies were peer-reviewed full-text articles (clinical trials, cohort studies, meta-analyses, preclinical experiments); excluded were case reports, letters, and non-English publications. Methodological quality was assessed using the Cochrane Risk of Bias tool for randomized controlled trials (RCTs), Newcastle-Ottawa Scale for observational studies, and SYRCLE tool for preclinical animal studies.

This review aims to systematically elucidate the emerging role of the gut-brain axis in the pathogenesis and treatment of ischemic stroke. We will first summarize how gut microbiota correlates with alterations to classical stroke risk factors, then delve into the molecular and cellular mechanisms underlying bidirectional gut-brain communication after stroke. Finally, we will comprehensively evaluate human-derived therapeutic options with clear differentiation from preclinical data, include large-sample-size clinical trials, discuss key confounders, expand limitations to address translational gaps and clinical heterogeneity, and discuss current challenges and future research directions, with the goal of informing novel, yet still preclinical, strategies for stroke prevention and therapy. We explicitly emphasize that robust large-scale multicenter randomized controlled trials are mandatory to validate all microbiota-targeted interventions before formal clinical guideline recommendations can be formulated.

## 2 The role of gut microbiota in stroke risk factors

**Figure 1:**
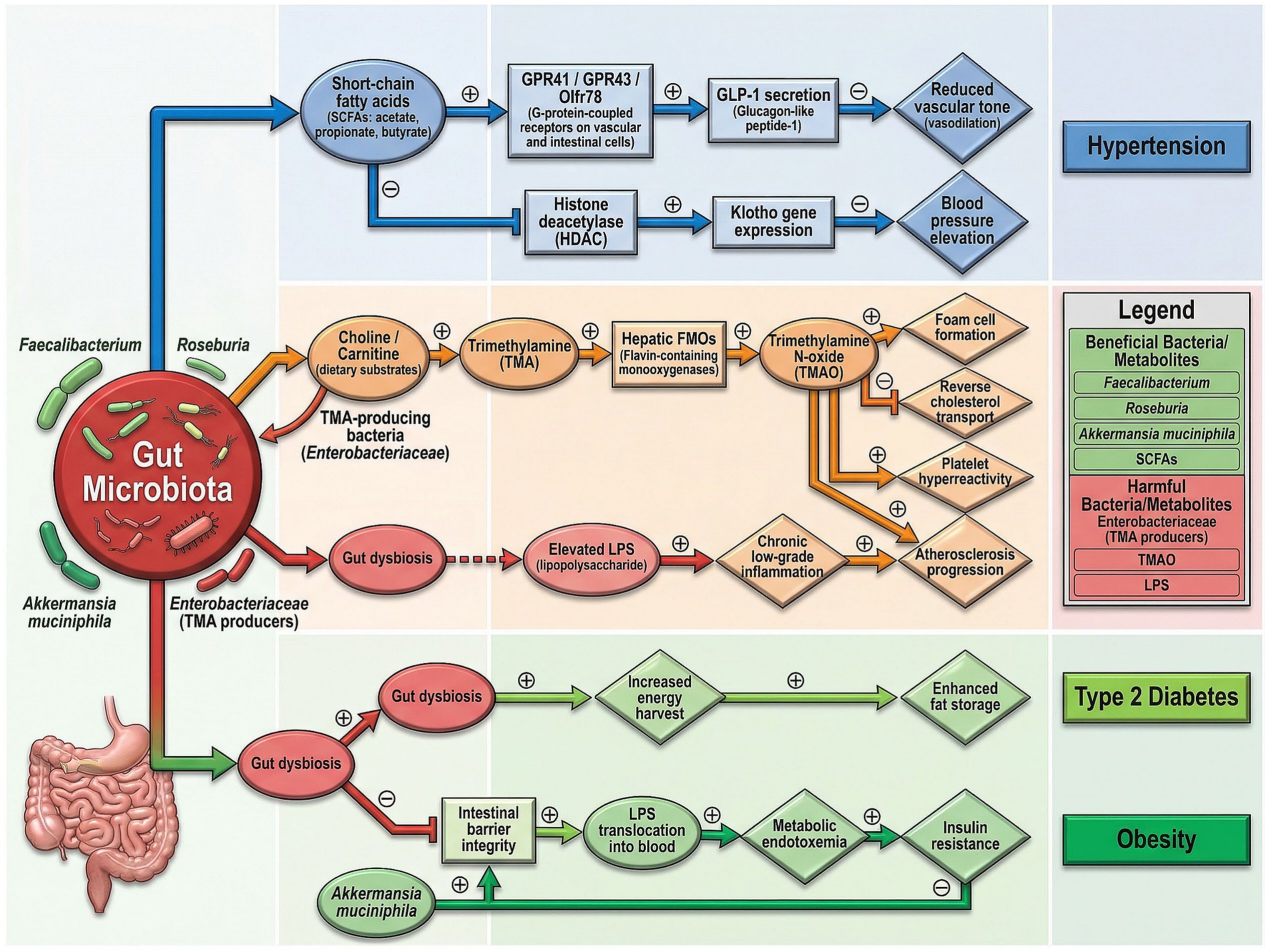
Gut microbiota modulates key risk factors for ischemic stroke via distinct molecular pathways. Schematic illustration of the specific molecular mechanisms by which gut microbiota and their bioactive metabolites regulate the four major stroke risk factors (hypertension, atherosclerosis, type 2 diabetes, and obesity). Beneficial microbial metabolites (e.g., short-chain fatty acids, SCFAs) and commensal bacteria (e.g., *Akkermansia muciniphila*, SCFA-producing bacteria) exert protective effects by inhibiting pathological processes (⊖), while harmful metabolites (e.g., trimethylamine N-oxide, TMAO; lipopolysaccharide, LPS) and dysbiotic microbiota promote the development of risk factors (⊕). Key molecular targets, signaling pathways, and pathological consequences are annotated for each risk factor branch, with representative beneficial and harmful bacterial strains listed in the legend. GPR, G protein-coupled receptor; GLP-1, glucagon-like peptide-1; FMOs, flavin monooxygenases; HDAC, histone deacetylase.

Before discussing the direct impact of gut microbiota on the acute pathological processes of ischemic stroke, it is essential to recognize its fundamental role in the development and progression of multiple key stroke risk factors (Figure 1). Across all human observational studies evaluating gut microbiota and stroke risk factors, reported associations remain correlational; causal inference is only supported by mechanistic preclinical animal experiments. Accumulating evidence indicates that gut microbiota dysbiosis is not merely an accompanying phenomenon but correlates with alterations to the pathophysiology of conditions such as hypertension, atherosclerosis, diabetes, and obesity by modulating host metabolic and immune homeostasis. Thus, it plays a critical “upstream” role in stroke pathogenesis supported primarily by preclinical causal data.

### 2.1 Hypertension

Across human observational studies evaluating gut microbiota and hypertension, reported associations remain correlational; causal inference is only supported by mechanistic preclinical animal experiments. Hypertension is one of the most potent and modifiable risk factors for ischemic stroke. Gut microbiota primarily is associated with blood pressure regulation via SCFAs—such as acetate, propionate, and butyrate—produced from the fermentation of dietary fibers (15). SCFAs exert broad physiological effects by activating G protein-coupled receptors (e.g., GPR41, GPR43, and Olfr78) and inhibiting histone deacetylases. For instance, SCFA activation of GPR43 promotes the secretion of glucagon-like peptide-1 (GLP-1) by intestinal endocrine cells, which subsequently modulates vascular tone via the GLP-1 receptor pathway. Inhibition of histone deacetylases can upregulate the expression of antihypertensive genes (e.g., Klotho) in the kidneys. Specifically, butyrate has been shown in preclinical models to promote the differentiation and function of regulatory T cells (Tregs), suppressing inflammatory responses in the kidneys and blood vessels, thereby contributing to blood pressure reduction (16). Conversely, reduced SCFAs production due to gut dysbiosis correlates with attenuation of this anti-inflammatory protective effect in human cohorts. Additionally, gut microbiota influences host electrolyte metabolism and sodium/water balance, which may indirectly correlate with blood pressure regulation (17). Human studies show significant differences in gut microbial composition between hypertensive patients and healthy individuals. Beneficial SCFA-producing bacteria are often reduced (18).

### 2.2 Atherosclerosis

Across human observational studies evaluating gut microbiota and atherosclerosis, reported associations remain correlational; causal inference is only supported by mechanistic preclinical animal experiments. Atherosclerosis is the core pathological basis of large-vessel occlusive stroke. One of the most representative mechanisms by which gut microbiota is associated with the progression of atherosclerosis is the trimethylamine N-oxide (TMAO) pathway. Dietary nutrients such as choline, lecithin, and carnitine are metabolized by specific gut bacteria into trimethylamine (TMA), which is absorbed via the portal vein and converted into TMAO in the liver by flavin monooxygenases (FMOs) (19). Most human evidence is associative, while preclinical studies support causality. Elevated plasma TMAO correlates with elevated risk of atherosclerosis through multiple pathways. It inhibits reverse cholesterol transport, promotes macrophage foam cell formation, and enhances platelet reactivity to correlate with increased thrombotic risk (19, 20). A meta-analysis of >19,000 participants shows that individuals with plasma TMAO ≥5 μmol/L have a 2.1-fold higher stroke risk (HR = 2.1, 95% CI: 1.6–2.8) than those with levels <2 μmol/L. TMAO is an independent correlative biomarker of cardiovascular events including stroke (21). Beyond the TMAO pathway, chronic low-grade inflammation from gut dysbiosis also correlates with accelerated atherosclerosis in human cohorts.

### 2.3 Diabetes and obesity

Across human observational studies evaluating gut microbiota and type 2 diabetes/obesity, reported associations remain correlational; causal inference is only supported by mechanistic preclinical animal experiments. Type 2 diabetes and obesity are closely related metabolic disorders and significant risk factors for ischemic stroke. Gut microbiota is associated with the pathogenesis of these conditions by modulating energy harvest, insulin sensitivity, and systemic inflammation levels. In obesity, altered gut microbiota structure enhances energy extraction from the diet and promotes fat storage in the host (22). Furthermore, dysbiosis can compromise intestinal barrier function, leading to the translocation of microbial products such as bacterial lipopolysaccharide (LPS) into the circulation. This triggers “metabolic endotoxemia,” activating chronic low-grade systemic inflammation and correlating with the development of insulin resistance (23). Beneficial bacteria such as *Akkermansia muciniphila* improve gut barrier integrity. They alleviate metabolic endotoxemia and insulin resistance. They show anti-diabetic and anti-obesity potential in preclinical models (24). Most evidence is preclinical; human data remain associative.

In summary, gut microbiota correlates with the development and progression of key stroke risk factors—hypertension, atherosclerosis, diabetes, and obesity—by regulating immune responses, producing bioactive metabolites, and maintaining intestinal barrier integrity. This underscores the promise of maintaining a healthy gut microbial ecosystem or correcting dysbiosis through interventions as a prospective “upstream” strategy for stroke prevention, though all human supporting data remain correlative. For individuals, their inherent gut microbial profile may partially correlate with susceptibility to traditional stroke risk factors.

## 3 Pathophysiological bidirectional communication of the gut-brain axis after stroke

**Figure 2:**
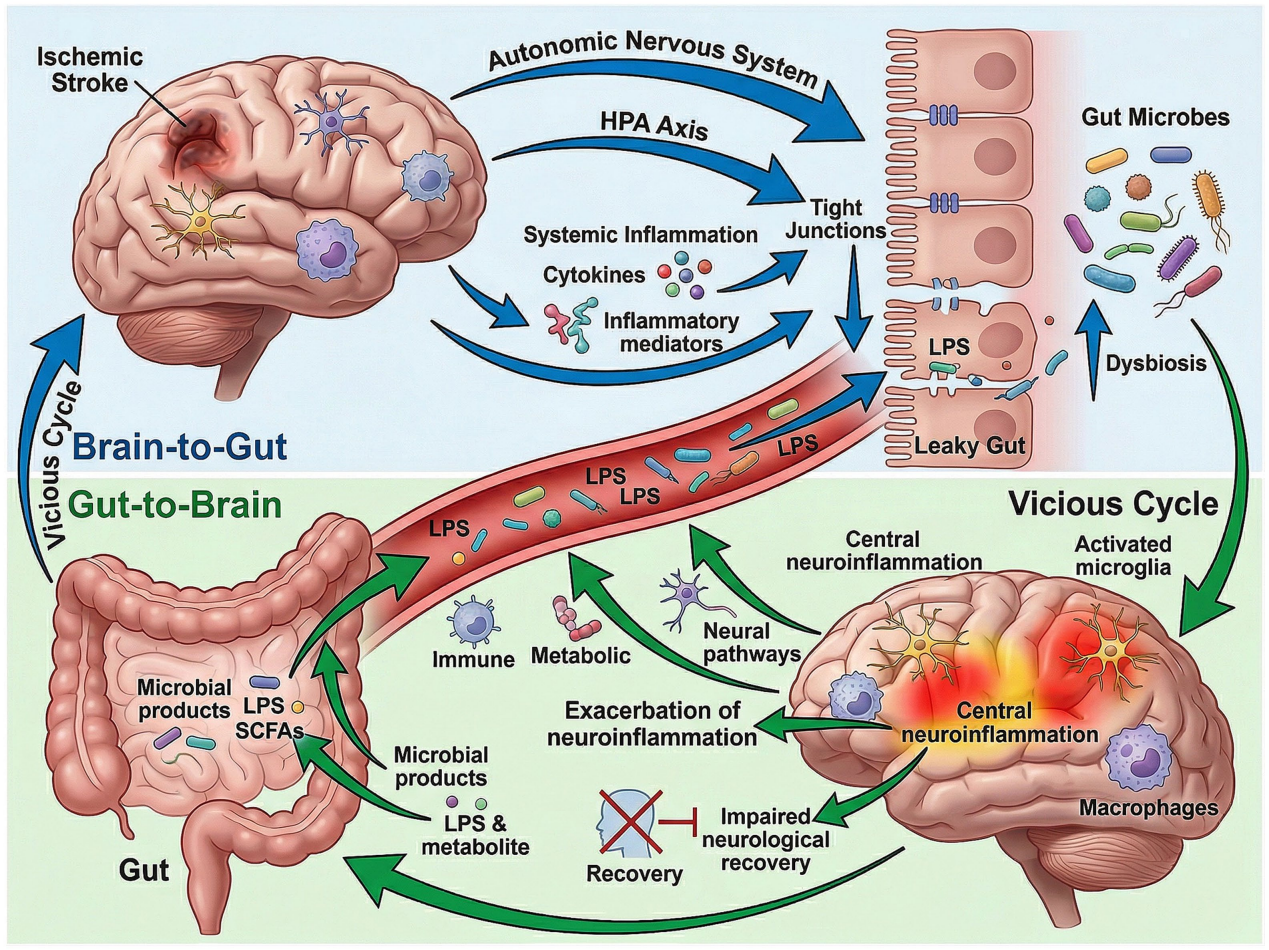
The vicious cycle of bidirectional gut-brain axis crosstalk following acute ischemic stroke: Schematic depicting the reciprocal pathological interaction between the brain and gut after ischemic stroke, which forms a self-reinforcing vicious cycle driving poor neurological outcomes. In the brain-to-gut direction (blue), cerebral ischemia triggers hyperactivation of the autonomic nervous system (ANS) and hypothalamic–pituitary–adrenal (HPA) axis, coupled with systemic inflammation, which impairs intestinal barrier integrity (leaky gut) and induces gut microbiota dysbiosis. This pathological state facilitates translocation of intestinal bacteria and their bioactive products into the systemic circulation, further amplifying systemic inflammatory responses. In the gut-to-brain direction (green), translocated microbial products and dysbiosis-derived metabolites act on the injured brain via immune, metabolic, and neural pathways, which exacerbates central neuroinflammation, enlarges cerebral infarct volume, and hinders post-stroke neural repair and functional recovery. Collectively, this bidirectional crosstalk perpetuates pathological damage across the brain-gut axis and contributes to adverse clinical outcomes in stroke patients.

Following the onset of ischemic stroke, the gut-brain axis does not remain static; instead, it rapidly initiates a complex bidirectional pathophysiological communication network (Figure 2). This process forms a vicious cycle: cerebral ischemia directly triggers intestinal dysfunction and gut microbiota dysbiosis (the “brain-to-gut” direction), while the altered gut environment, in turn, acts upon the brain through multiple pathways, exacerbating neuroinflammation and impeding repair (the “gut-to-brain” direction). A deep understanding of this bidirectional communication mechanism is crucial for developing innovative therapies targeting the entire course of stroke.

### 3.1 Brain-to-gut direction: impact of stroke on the gut environment and subsequent systemic inflammatory effects

An ischemic stroke event can rapidly exert profound effects on the distant gut through multiple mechanisms. First, stroke-induced significant elevation in sympathetic tone and overactivation of the hypothalamic–pituitary–adrenal (HPA) axis lead to reduced intestinal blood flow, inhibited gastrointestinal motility, and impaired mucosal barrier function (11, 25). This “leaky gut” state allows intestinal bacteria and their products, such as lipopolysaccharide (LPS), to translocate into the portal circulation and systemic system, triggering or exacerbating systemic inflammatory responses and sepsis risk, which correlate with poor outcomes in stroke patients (7, 12). Studies in animal models have confirmed that within hours after middle cerebral artery occlusion (MCAO), downregulation of intestinal tight junction proteins (e.g., Occludin, ZO-1), mucosal atrophy, and bacterial translocation can be observed (26). Studies in middle cerebral artery occlusion (MCAO) rat models have shown that as early as 6 h after ischemia, the expression of intestinal tight junction proteins (Occludin and ZO-1) decreases by 35–40%, accompanied by a 2.5-fold increase in intestinal mucosal permeability (measured by FITC-dextran leakage), and bacterial translocation to the mesenteric lymph nodes is detectable at 12 h (26). Second, systemic immunosuppression triggered by stroke also alters the local intestinal immune environment, further promoting gut microbiota dysbiosis and creating a microenvironment conducive to the growth of pathogenic bacteria (11).

### 3.2 Gut-to-brain direction: regulation of stroke outcome by the gut

Dysregulated gut microbiota and their metabolites correlate with the extent of post-stroke brain injury and functional recovery through three major pathways: immune, metabolic, and neural.

#### 3.2.1 Immune pathway

This is the most central mechanism. Translocation of microbial products (such as LPS) and dead bacteria through the “leaky gut” into the circulation activates systemic immune cells, thereby promoting the infiltration of pro-inflammatory lymphocytes (e.g., Th1 and Th17) into the brain. These pro-inflammatory lymphocytes primarily enter the central nervous system via impaired regions of the blood–brain barrier or the choroid plexus, synergizing with intracerebral microglia to exacerbate neuroinflammation. Meanwhile, gut microbiota-derived metabolites, such as SCFAs, can modulate the activation state of microglia. Studies show that mice lacking normal gut microbiota (germ-free mice) or those with antibiotic-depleted microbiota exhibit defective microglia, attenuated neuroinflammation, and reduced infarct volume after stroke (14, 27). Specifically, germ-free mice have a 30% smaller infarct volume and 25% lower levels of pro-inflammatory cytokines (TNF-α, IL-6) in the ischemic penumbra compared to conventional mice 24 h after MCAO (27). Specific bacterial populations, such as those producing indole-3-propionic acid, can limit central nervous system inflammation by activating the aryl hydrocarbon receptor (AhR), exerting neuroprotective effects (28).

#### 3.2.2 Metabolic pathway

Metabolites derived from gut microbiota directly participate in the pathophysiological regulation after stroke in preclinical models; human data only support correlative associations. As previously mentioned, the harmful metabolite TMAO not only correlates with accelerated atherosclerosis but also enhances platelet reactivity, increasing the risk of post-stroke thrombosis (20). Conversely, beneficial metabolites like SCFAs (particularly butyrate) demonstrate anti-inflammatory and neuroprotective properties. They can enhance blood–brain barrier integrity, promote the polarization of microglia toward an anti-inflammatory phenotype, and support the expression of brain-derived neurotrophic factor (BDNF), thereby benefiting neural repair (16, 29). Butyrate exerts neuroprotective effects through multiple pathways: it upregulates the expression of tight junction proteins (Claudin-5, Occludin) in brain microvascular endothelial cells to enhance blood–brain barrier integrity; it promotes the polarization of microglia to the M2 anti-inflammatory phenotype by inhibiting histone deacetylase 1; and it increases the expression of brain-derived neurotrophic factor (BDNF) in the hippocampus by activating the GPR43-PI3K-AKT pathway, thereby promoting neural repair (16, 29, 30).

#### 3.2.3 Neural pathway

The gut microbiota communicates with the brain via the vagus nerve—a direct anatomical pathway forming a core neural gut-brain communication circuit. Gut microbiota and their metabolites regulate enterochromaffin cell synthesis and secretion of neuroactive signaling molecules, which modulate vagal afferent neuronal firing to transmit peripheral gut signals centrally. Key microbiota-derived bioactive compounds that modulate enteric neuron and vagal afferent activity include: tryptophan metabolites (indole derivatives), gamma-aminobutyric acid (GABA), serotonin precursors, and SCFAs. Enterochromaffin cells produce >90% of systemic serotonin; microbial SCFAs stimulate serotonin biosynthesis via upregulating tryptophan hydroxylase 1, increasing serotonin release into the lamina propria to activate vagal afferent nerve terminals. Microbial tryptophan catabolites bind aryl hydrocarbon receptors on enteric neurons to suppress pro-inflammatory neural signaling, while GABA-producing commensal bacteria directly modulate inhibitory enteric neural transmission. Collectively, these gut-derived signals propagate along the vagus nerve to the brainstem nucleus tractus solitarius, altering central autonomic, inflammatory, and neurotrophic signaling cascades post-stroke. Studies indicate that vagus nerve stimulation (VNS) has a certain therapeutic effect in stroke rehabilitation, partly through its modulation of gut function and microbiota composition, revealing the existence of a “brain-gut-brain” feedback loop (31). A simplified schematic of this gut-vagus-brain neural circuitry is integrated into revised Figure 2 to balance coverage of immune, metabolic, and neural gut-brain communication pathways.

**Figure 3:**
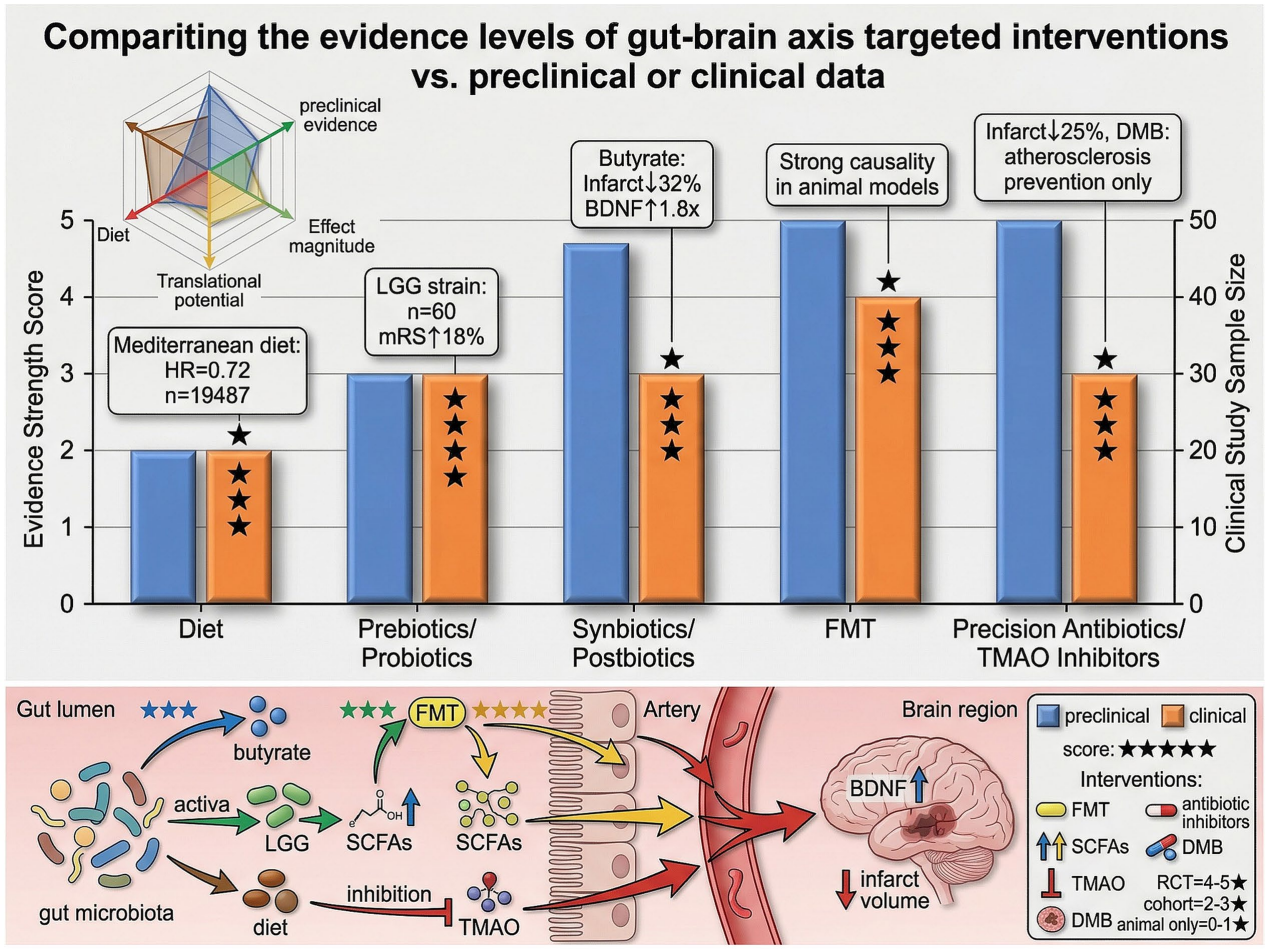
Comparison of preclinical and clinical evidence levels for gut-brain axis-targeted interventions in ischemic stroke. Grouped bar chart showing the evidence strength (0–5 scale, 5 = strongest) of five major gut-brain axis-targeted intervention strategies for ischemic stroke, with separate quantification for preclinical (blue) and clinical (orange) evidence. The left vertical axis represents evidence strength score, and key clinical/preclinical data (e.g., sample size, hazard ratio (HR), infarct volume reduction rate) are annotated above each bar. Evidence strength was graded based on study type (randomized controlled trial, RCT; cohort study; animal experiment), sample size, and statistical significance: 4–5 = strong evidence, 2–3 = moderate/emerging evidence, 0–1 = preliminary/exploratory evidence. Stars (★) on the right of each bar represent the overall evidence grade (5 stars = strongest). FMT, fecal microbiota transplantation; SCFAs, short-chain fatty acids; TMAO, trimethylamine N-oxide; DMB, 3,3-dimethyl-1-butanol; mRS, modified Rankin Scale; NIHSS, National Institutes of Health Stroke Scale.

In summary, the post-stroke bidirectional communication along the gut-brain axis constitutes a dynamic and complex vicious cycle. The initial brain injury disrupts gut homeostasis, while subsequent gut microbiota dysbiosis and barrier dysfunction, in turn, amplify neuroinflammation, impede tissue repair, and ultimately lead to worse neurological outcomes. Intervening at any node within this cycle may represent an effective preclinical strategy to improve stroke prognosis, though human clinical validation remains incomplete (Figure 3).

## 4 Novel therapeutic strategies targeting the gut-brain axis for stroke

Based on the in-depth understanding of the critical role of the gut-brain axis in the pathophysiology of ischemic stroke, a series of innovative therapeutic strategies aimed at modulating the gut microbial ecosystem to improve stroke outcomes are being extensively explored. These interventions span the entire course of stroke management—from prevention and acute-phase treatment to long-term rehabilitation—with the core objectives of restoring a healthy microbial composition, reinforcing intestinal barrier function, and increasing the production of beneficial microbial metabolites, thereby breaking the aforementioned vicious cycle (Table 1).

**Table 1:** Gut-brain axis-targeted therapeutic strategies for ischemic stroke management: mechanisms, agents and evidence grading.

Intervention strategy	Core mechanisms of action	Representative agents/methods	Evidence level and translational remarks
Dietary modification	Shapes eubiotic gut microbiota composition; enhances production of protective microbial metabolites (e.g., short-chain fatty acids, SCFAs); reinforces intestinal barrier integrity	High-fiber diet, Mediterranean diet (rich in fruits, vegetables, whole grains and olive oil)	Preclinical: Strong (consistent neuroprotective effects in rodent stroke models); Clinical: Moderate (support from large prospective cohort studies); serves as a foundational, accessible first-line strategy for stroke prevention and secondary intervention
Prebiotics/Probiotics	Prebiotics: Selectively promote the colonization and proliferation of beneficial commensal bacteria; Probiotics: Directly supplement live beneficial microbes to modulate host immune responses and restore intestinal barrier function	Prebiotics: Inulin, fructooligosaccharides (FOS); Probiotics: *Lactobacillus* spp., *Bifidobacterium* spp.	Preclinical: Strong (attenuates neuroinflammation and improves functional recovery in animal models); Clinical: Moderate (mixed results from small randomized controlled trials (RCTs)); critical unmet need for standardization of strains, dosages and treatment durations
Synbiotics/Postbiotics	Synbiotics: Synergistic combination of prebiotics and probiotics to enhance microbial colonization and efficacy; Postbiotics: Deliver inactivated microbial cells, microbial components or bioactive metabolites to exert protective effects without live bacteria-associated risks	Synbiotics: Combined prebiotic-probiotic formulations; Postbiotics: SCFAs (sodium butyrate), microbial exopolysaccharides, heat-inactivated probiotic lysates	Preclinical: Emerging (promising neuroprotective effects in rodent stroke models); Clinical: Preliminary (no large-scale human studies); postbiotics hold high translational potential due to superior stability and safety profiles
Fecal microbiota transplantation (FMT)	Holistically reconstructs a healthy gut microbial ecosystem by transferring fecal microbiota from healthy donors; reverses stroke-induced gut dysbiosis and restores brain-gut axis homeostasis	Allogeneic FMT (fecal microbiota from healthy human donors), syngeneic FMT (rodent models)	Preclinical: Strong (provides causal evidence for microbial modulation of stroke outcomes in animal models); Clinical: Exploratory (no large RCTs); major barriers include donor screening standardization, preparation protocols and long-term safety assessment
Targeted metabolic pathway inhibition/Precision antibiotics	Precision antibiotics: Selectively eliminate pro-inflammatory pathogenic bacteria without disrupting beneficial commensal microbiota; Metabolic inhibitors: Block production of harmful microbial metabolites to abrogate their pro-atherosclerotic and pro-thrombotic effects	Precision antibiotics: Rifaximin (non-absorbable); Metabolic inhibitors: 3,3-dimethyl-1-butanol (DMB, TMAO synthesis inhibitor)	Preclinical: Moderate (efficacious in reducing atherosclerosis and infarct volume in animal models); Clinical: Preliminary (no stroke-specific human studies); primarily targeted for upstream stroke prevention via modulation of cardiovascular risk factors

SCFAs, short-chain fatty acids; FOS, fructooligosaccharides; FMT, fecal microbiota transplantation; TMAO, trimethylamine N-oxide; DMB, 3,3-dimethyl-1-butanol. Evidence grading is based on study type, sample size, statistical significance and reproducibility of results, consistent with Stroke and American Heart Association (AHA) evidence classification criteria. All strategies aim to disrupt the post-stroke brain-gut axis vicious cycle by restoring gut microbial homeostasis, enhancing intestinal barrier function and increasing the production of protective microbial metabolites.

### 4.1 Dietary interventions

Dietary modulation is the most fundamental and accessible approach. It rapidly shapes gut microbial community structure and function. High-fiber diets effectively increase beneficial SCFA production, especially butyrate.

(1) Preclinical evidence: Animal studies show fermentable fiber or high-fiber diet supplementation elevates circulating SCFA levels. It reduces cerebral infarct volume and improves neurological deficits after stroke by regulating immune cell function (16, 29). MCAO mice fed a high-fiber diet (15% cellulose) for 4 weeks before ischemia show 2.3-fold higher plasma butyrate, 32% smaller infarct volume, and 40% better neurological deficit scores than controls (16, 29).

(2) Clinical evidence (large cohort): The Mediterranean diet (rich in fruits, vegetables, whole grains, olive oil) links to a healthier gut microbiota and lower cardiovascular risk. It partly acts via the gut-brain axis. A prospective cohort of 19,487 UK Biobank adults followed for 11 years shows higher Mediterranean diet adherence correlates with lower stroke risk (HR = 0.72, 95% CI: 0.61–0.85). Mediation analysis shows 28% of the protective effect comes from increased SCFA-producing bacteria (e.g., Roseburia, Faecalibacterium) (32). Key confounding limitation: This primary UK Biobank analysis did not fully adjust for household income, educational attainment, and baseline dietary access as socioeconomic covariates; residual unmeasured socioeconomic confounding may attenuate or exaggerate the reported protective effect size. Future prospective dietary-microbiome stroke cohorts must incorporate comprehensive socioeconomic, medication, and comorbidity adjustment in multivariable regression models.

### 4.2 Prebiotics and probiotics

Prebiotics (e.g., fructooligosaccharides, inulin) are indigestible dietary components. They selectively promote beneficial bacteria growth for indirect microbial intervention. Probiotics are live microorganisms that confer health benefits.

(1) Preclinical evidence: Specific probiotic strains (e.g., Lactobacillus, Bifidobacterium) attenuate neuroinflammation, protect the BBB, and improve neurological recovery in stroke animal models (33).

(2) Clinical evidence: Clinical translation is limited and inconsistent. A 2021 single-center randomized trial (*n* = 60) shows 4-week *Lactobacillus rhamnosus* GG supplementation (10^10^ CFU/day) started within 72 h of acute ischemic stroke improves 14-day NIHSS scores. It increases favorable 3-month modified Rankin Scale rates by 18% (*p* = 0.038) (34). Key confounding limitation: This trial did not adjust for baseline statin, antiplatelet, or hypoglycemic medication use at enrollment; these medications directly alter gut microbial composition and may represent major unmeasured confounders biasing the observed treatment effect. Future probiotic stroke RCTs must mandate baseline collection and multivariable adjustment for all chronic cardiovascular and metabolic medications. Clinical evidence strength is low due to small sample size. Larger multicenter sham-controlled trials are needed to confirm efficacy.

### 4.3 Synbiotics and postbiotics

Synbiotics refer to synergistic combinations of probiotics and prebiotics, designed to simultaneously supplement live bacteria and provide nutrients for their colonization and growth in the gut. Postbiotics include inactivated microbial cells, microbial components, and their metabolites (e.g., SCFAs, exopolysaccharides). Postbiotics circumvent biological safety concerns related to live bacteria, such as survival rates and translocation risks, offering a more stable and controllable intervention option, and their therapeutic value is gaining increasing attention (35). For example, direct supplementation with SCFAs like butyrate has successfully replicated some neuroprotective effects of high-fiber diets in animal models (29). In a rat model of MCAO, intraperitoneal injection of sodium butyrate (100 mg/kg/day) for 7 days after ischemia increased BDNF expression in the ischemic penumbra by 1.8 fold, reduced microglial activation by 40%, and improved motor function recovery by 35% compared to the saline group (29).

### 4.4 Fecal microbiota transplantation

Fecal microbiota transplantation (FMT) involves transferring processed fecal microbiota from healthy donors into a patient’s gut to reconstruct a healthy microbial ecosystem. In stroke research, FMT provides direct causal evidence: transplanting fecal microbiota from stroke-prone hypertensive rats into normotensive rats exacerbates post-stroke brain injury, whereas microbiota from healthy donors significantly improves stroke outcomes (36). Clinical application of FMT in stroke patients remains exploratory and awaits larger randomized controlled trials.

### 4.5 Other intervention strategies: precision antibiotics and targeted metabolic pathway drugs

Other approaches show potential. In specific contexts, precision antibiotics eliminate pro-inflammatory pathogens to benefit stroke outcomes. Caution is needed to avoid dysbiosis risk.

(1) Preclinical evidence: In stroke mice with Enterobacteriaceae-dominated dysbiosis, targeted rifaximin (non-absorbable antibiotic) reduces Enterobacteriaceae by 90%. It decreases plasma TNF-*α* by 35% and infarct volume by 25% (14, 37). Broad-spectrum antibiotics (e.g., ampicillin-sulbactam) increase infarct volume by 15% by disrupting beneficial microbiota (37). Targeting harmful metabolic pathways (e.g., DMB inhibiting TMAO-producing enzymes) reduces atherosclerosis in animal models. It offers new stroke prevention avenues (38).

(2) Clinical evidence: No large-sample stroke-specific clinical trials exist. Preliminary cardiovascular trials (n > 500) show DMB safety but no long-term stroke outcome data.

In summary, targeting the gut-brain axis opens a promising new frontier for stroke prevention and treatment. Current research is shifting from describing correlations to exploring causal mechanisms and testing the efficacy of interventions. Future directions lie in achieving precision microbial medicine—tailoring the most effective microbiota-based interventions according to an individual’s microbiome profile, genetic background, and stroke subtype—to translate this revolutionary paradigm into tangible clinical benefits.

## 5 Challenges and future directions

Although targeting the gut-brain axis presents exciting prospects for the prevention and treatment of ischemic stroke, this field remains in its early developmental stages and faces a series of critical challenges in translating basic research into clinical applications. Clearly recognizing these challenges and planning future research directions accordingly are essential for advancing the maturity of this revolutionary paradigm.

### 5.1 Elucidating causality beyond correlation

Most current human studies primarily demonstrate correlations between gut microbial composition and stroke risk or outcomes, but definitive causal relationships require further validation. Although research such as fecal microbiota transplantation (FMT) provides strong causal evidence in animal models (36), establishing the direct pathogenic or protective roles of specific bacterial strains or their metabolites in humans remains complex. Future studies should integrate multi-omics technologies—including metagenomics, metabolomics, and proteomics—combined with germ-free animals and gnotobiotic models colonized with specific microbiota to precisely dissect the complete causal chain: “which specific species—through which metabolites/signals—how they affect host pathways—and what outcomes in stroke pathology” (14, 27).

### 5.2 Translational gaps between preclinical and clinical research

Preclinical studies are predominantly conducted in young, genetically homogeneous rodents under controlled conditions, which poorly recapitulate the elderly, comorbid, and medicated human stroke population. Most preclinical findings have not been replicated in large-sample human trials, creating a substantial translational bottleneck. Neuroprotective effects observed in animal models often do not translate to clinical efficacy due to species differences, variable intervention timing, and lack of clinically relevant endpoints.

### 5.3 Heterogeneity of human clinical trials

Human trials exhibit extreme heterogeneity in subject enrollment (age, stroke subtype, comorbidities), intervention protocols (strain, dose, duration of probiotics; FMT donor selection), outcome measures (NIHSS, mRS, inflammatory markers), and follow-up periods. This heterogeneity prevents direct cross-trial comparison and meta-analysis, weakening the level of clinical evidence. Small sample sizes (most <100) further limit statistical power and generalizability.

### 5.4 Individual variability and precision medicine

The gut microbiome exhibits high inter-individual heterogeneity, influenced by factors such as age, sex, genetic background, geographic environment, dietary habits, and comorbidities (e.g., diabetes, hypertension) (39). This heterogeneity may lead to divergent responses to the same intervention in different individuals. For instance, age-related dysbiosis in the elderly may result in responses to certain probiotic interventions that differ from those in younger individuals (32). The future direction lies in advancing toward precision microbial medicine, which involves analyzing patients’ microbiome profiles to identify biomarkers that can predict treatment response or prognosis, thereby tailoring the most effective microbiota-modulating strategies for specific subgroups of stroke patients.

### 5.5 Optimization of intervention timing and duration

The timing and duration of interventions are critical variables influencing efficacy, yet no unified standards currently exist. For stroke prevention, long-term lifestyle or dietary interventions may be key. In the acute phase of stroke, the goal is to rapidly stabilize the intestinal barrier, suppress the overgrowth of harmful bacteria, and reduce the production of detrimental metabolites. During the recovery phase, interventions may focus on persistently modulating the microbiota to promote neuroplasticity and functional reconstruction (29, 34). Future research should design rigorous preclinical and clinical studies to systematically compare the effects of interventions initiated at different time windows (prevention, acute phase, recovery) to determine optimal therapeutic strategies.

### 5.6 Standardization and safety concerns

Another major challenge in this field is the lack of standardization. Whether for probiotics (strain, viable count, formulation), synbiotics, or FMT (donor screening, preparation protocols, quality control standards), there are substantial variations in composition and potency, making it difficult to compare and replicate study results (40). Furthermore, the long-term safety of invasive therapies like FMT requires evaluation through large-scale studies, particularly regarding potential risks of transmitting unknown pathogens or inducing long-term metabolic abnormalities. Establishing internationally recognized standardized operating procedures and rigorous safety monitoring systems is a prerequisite for clinical translation (40).

### 5.7 Development and validation of biomarkers

Developing gut microbial features or circulating metabolites into clinically applicable biomarkers holds significant potential. For example, plasma TMAO levels have been explored for risk stratification of cardiovascular events (21). In a recent prospective cohort, reduced gut microbiota-derived indole-3-propionic acid was independently associated with increased stroke recurrence risk (HR = 2.31, 95% CI: 1.45–3.68) (41). Multi-omics approaches—such as metagenomics for analyzing gut microbial gene composition, metabolomics for detecting microbial metabolites, and proteomics for focusing on host-microbiota protein interactions—should be leveraged in future research to discover and validate microbial or metabolite biomarkers that can specifically predict stroke risk, acute injury severity, or long-term recovery potential. This will not only improve stratified management of stroke but also provide a basis for personalized treatment.

**Figure 4:**
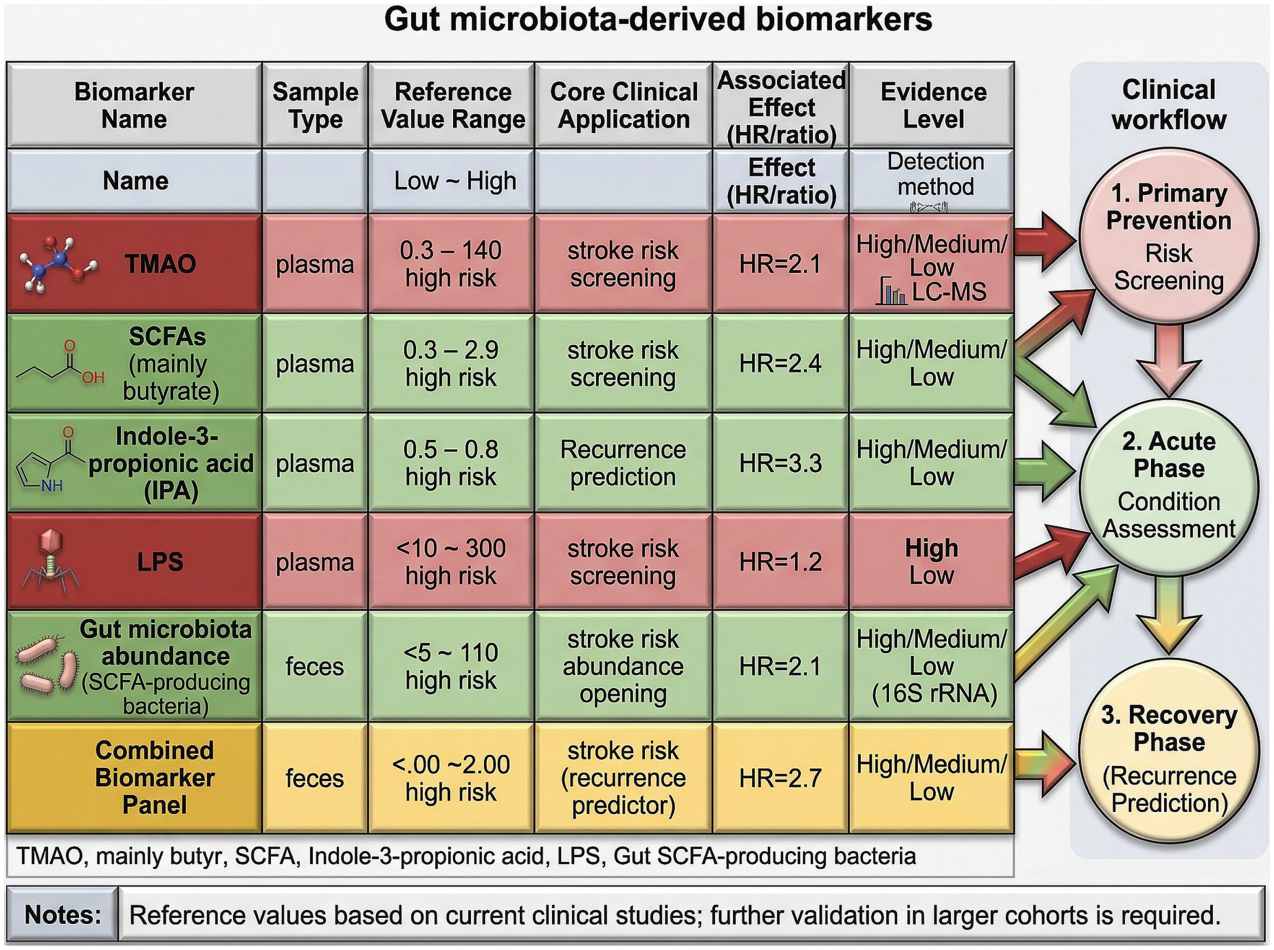
Clinical application value of gut-brain axis-related biomarkers in ischemic stroke. Tabular schematic summarizing the core clinical characteristics of gut-brain axis-derived biomarkers for ischemic stroke, including biomarker name, detection sample, reference value range, primary clinical application scenario, associated effect size (hazard ratio, HR), and evidence level. Biomarkers are color-coded by their functional role: red for risk-associated biomarkers (TMAO, LPS), green for protective biomarkers (SCFAs, IPA, SCFA-producing bacteria abundance), and yellow for the composite biomarker panel. Evidence level is classified as high (meta-analysis/large prospective cohort), moderate (small prospective cohort/RCT), or low (preclinical/observational study). Key detection methods and clinical application nodes (primary prevention, acute phase assessment, recurrence prediction) are annotated, with reference values derived from human clinical studies. TMAO, trimethylamine N-oxide; SCFAs, short-chain fatty acids; IPA, indole-3-propionic acid; LPS, lipopolysaccharide; LC–MS, liquid chromatography-mass spectrometry; 16S rRNA, 16S ribosomal ribonucleic acid.

Despite promising correlative cohort data for TMAO, SCFAs, IPA, and LPS as gut-brain axis biomarkers, multiple practical barriers prevent routine clinical adoption. First, published reference concentration ranges lack external validation across independent multiethnic stroke cohorts, limiting universal cut-off value implementation. Second, none of the analytes are stroke-specific; TMAO, LPS, and SCFA levels are altered in heart failure, chronic kidney disease, and metabolic syndrome, restricting diagnostic specificity for cerebrovascular events. Third, detection platforms including liquid chromatography-mass spectrometry (LC–MS) and 16S rRNA gene sequencing are high-cost laboratory research assays with long sample turnaround times, absent standardized cross-laboratory calibration protocols, and no universal medical insurance reimbursement pathways in most healthcare systems. Collectively, these limitations mean all gut-microbiota-derived biomarkers discussed herein remain research-use-only at present; substantial prospective validation, assay standardization, and health economic evaluation are required before bedside clinical deployment. These barriers are integrated into revised Figure 4 annotations to clearly distinguish research biomarkers from clinically ready diagnostic tools.

### 5.8 Key confounders: comorbidities, medications, and socioeconomic factors

Comorbidities (hypertension, diabetes, chronic kidney disease, cardiovascular disease) alter gut microbiota composition independently of stroke, confounding the observed microbiota-stroke associations. Most stroke patients are on multiple medications (antiplatelets, statins, antihypertensives, antibiotics), which directly modulate gut microbiota and may mask or amplify intervention effects. Socioeconomic factors (dietary quality, access to healthcare, living environment) drive consistent differences in gut microbiota across populations, introducing unmeasured confounding in observational studies. Few studies adjust for these covariates in multivariable models, limiting causal inference.

## 6 Conclusion

Ischemic stroke research is undergoing a paradigm shift. Stroke, once a localized cerebrovascular event, is now a systemic multi-organ interaction disease. The gut-brain axis is central to this new paradigm. This review systematically explains how gut microbiota and metabolites regulate stroke risk factors, acute injury, and long-term neural repair via immune, metabolic, and neural pathways. Bidirectional gut-brain axis communication forms a vicious cycle: brain injury causes gut barrier dysfunction and dysbiosis. The imbalanced gut then worsens neural damage and hinders recovery via inflammation and metabolic changes (14, 27, 33).

Elucidating these mechanisms provides opportunities for novel stroke prevention and treatment strategies (29, 33, 35). Targeting gut microbiota via diet, probiotics, prebiotics, postbiotics, or FMT is an innovative therapeutic approach. These interventions enhance intestinal barrier integrity, increase beneficial metabolites (e.g., SCFAs), and reduce pro-inflammatory mediators and harmful metabolites (e.g., TMAO). They modulate central pathology from the periphery.

Translating this concept to clinical practice faces major limitations. Clinical evidence strength is low, with most data from small early-phase studies. Overinterpretation should be avoided. Current research relies heavily on animal models and correlative analyses. Future work must establish human causality and address translational gaps, clinical heterogeneity, individual variability, intervention timing, protocol standardization, confounders (comorbidities, medications, socioeconomic status), and long-term safety.

Future research should use multi-omics and bioinformatics to dissect microbiota-host interaction mechanisms. Well-designed large-sample prospective clinical trials are essential. This will enable microbiome-based precision stroke medicine.

In summary, exploring the gut-brain axis in ischemic stroke enriches pathophysiological understanding and opens a promising therapeutic frontier. Bidirectional gut-brain axis vicious cycling is central to stroke pathophysiology. Gut-targeted interventions show multi-stage therapeutic potential. This field provides a new interdisciplinary paradigm for neurological disease prevention and treatment. It offers insights for other central nervous system disorders (e.g., Alzheimer’s disease). Further research will deliver novel, effective stroke strategies to improve long-term prognosis and quality of life.
